# Surgical management in a rare case of basaloid squamous cell carcinoma of the maxilla

**Published:** 2015

**Authors:** I Tuhar, D Zamfirescu, D Slăvescu, A Frunză, I Lascăr

**Affiliations:** * Clinic of Plastic Surgery and Reconstructive Microsurgery, “Carol Davila” Military Emergency Hospital, Bucharest, Romania; ** Clinic of Plastic Surgery and Reconstructive Microsurgery, Bucharest Emergency Hospital, Bucharest, Romania; *** “Carol Davila” University of Medicine and Pharmacy Bucharest, Romania

**Keywords:** basaloid squamous cell carcinoma, squamous cell carcinoma

## Abstract

Basaloid squamous cell carcinoma (BSCC) is a rare and aggressive version of squamous cell carcinoma (SCC) that preferentially occurs in the upper aerodigestive tract. Since the first description by Wain SL et al., in 1986, only 21 cases with BSCC in the nasal cavity or in the paranasal cavity have been reported in the English literature. We present a case of BSCC arising in a paranasal sinus, a 51-year-old male patient with four months history of right cheek swelling and unilateral nasal obstruction, who underwent an operation and postoperative radiotherapy. Clinical, pathological and surgical findings in this case are presented along with a brief discussion of literature.

## Introduction

Wain SL et al [**1**] first proposed in 1986 that basaloid squamous cell carcinoma (BSCC) was a distinct variant of squamous cell carcinoma (SCC). Basaloid squamous cell carcinoma is a high-grade and aggressive variant of squamous cell carcinoma that is most commonly found in the upper aerodigestive tract [**1**–**14**]. In 1991, the World Health Organization included this tumor in the revised classification for the upper respiratory tract and ear [**15**].

The most common sites of occurrence in the upper aerodigestive tract are the oral cavity, the larynx, the hypopharynx, the pyriform sinus, the tonsils and the base of tongue. The other less frequently affected sites are nose, paranasal sinus, gingiva, external ear, sub-mandibular region, esophagus, lung, anus, vulva, vagina and the uterine cervix [**2**,**16**].

So far, only 21 cases of BSCC of the nose and paranasal sinuses have been reported in the English literature [**2**,**7**–**19**].

In case of major defects involving the midface of posttraumatic etiology, post-combustion or the outcome of an oncological resection, due to the volume and extent of the structure to be rebuilt, the use of loco-regional flaps is not enough and the most effective method is the use of microsurgical transferred flaps.

In case of microsurgical reconstruction through the use of free flaps, the musculocutaneous rectus abdominis flap is frequently used. This flap has some advantages: long pedicle, anatomically constant that can be anastomosed to facial vessels (artery, vein) or transverse facial vessels; could be accommodated for complex defects that require the bulk of soft tissue; decreasing possible outbreaks of osteitis.

## Patient, Methods and Results

**Fig. 1:**
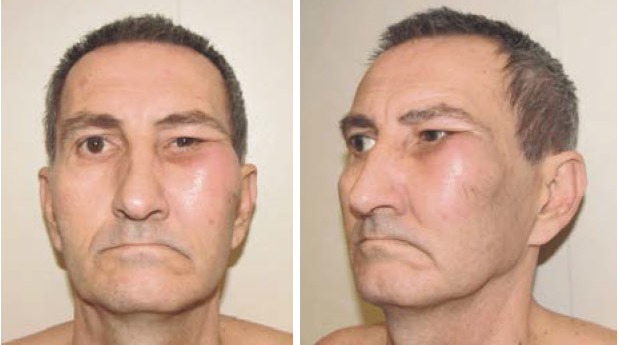
A 51-year-old male patient with four months history of right cheek swelling and unilateral nasal obstruction

A rare case of tumor investigated and operated in our department is presented. A 51-year-old male patient was admitted in our clinic with four months history of right cheek swelling (**Fig. 1**) and unilateral nasal obstruction.

**Fig. 2:**
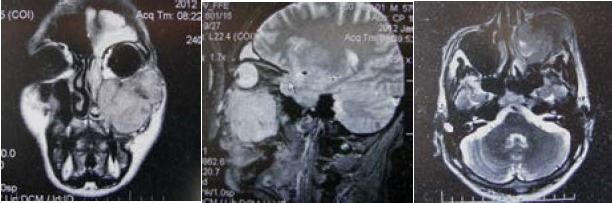
MRI of the left maxillary sinus showing that the tumor invaded the nasal cavity and the maxillary sinus

The clinical examination revealed a mass in the left nasal cavity and paranasal sinus. The past medical history was unremarkable. There was no significant history of smoking or alcohol consumption. There was no evidence of either a lymphatic or a distant metastasis at the time of diagnosis. Magnetic resonance imaging (MRI) demonstrated a tumor involving the right maxillary sinus (**Fig. 2**).

**Fig. 3 a,b,c:**
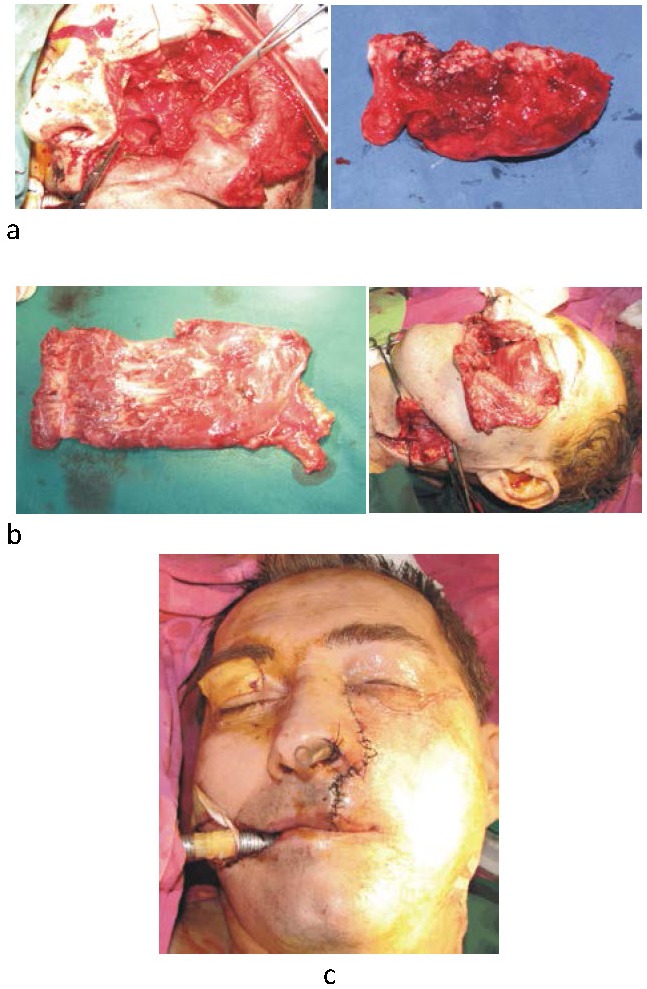
Tumoral excision and reconstruction of the defect with a rectus abdominis free flap

The patient was treated by a wider surgical excision of the mass (hemiresection of the left maxilla) and reconstruction with a rectus abdominis free flap (**Fig. 3 a,b,c**). The inferior epigastric vascular pedicle of the rectus abdominis was anastomosed to the facial vessels. The surgery was followed by radiotherapy.

(a) Excision of the tumor; (b) Rectus abdominis muscle used as a free flap for the reconstruction of the excisional defect; (c) Immediate postoperative result.

The pathologic microscopic examination identified the tumoral mass as an infiltrative basal cell carcinoma, with no tumoral elements left in place.

**Fig. 4:**
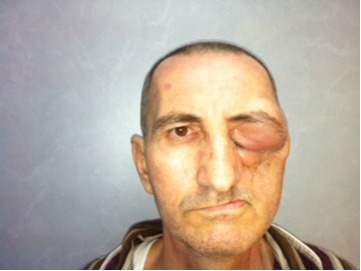
Result: 5 months after surgery

However, at 5 months follow-up, the patient presented with an edema of the left eyelids and inability to open them (**Fig. 4**); the MRI confirmed a tumoral mass infiltrating the extraocular fat and the right inferior muscle of the left ocular globe, with an extension to the frontal sinus.

**Fig. 5:**
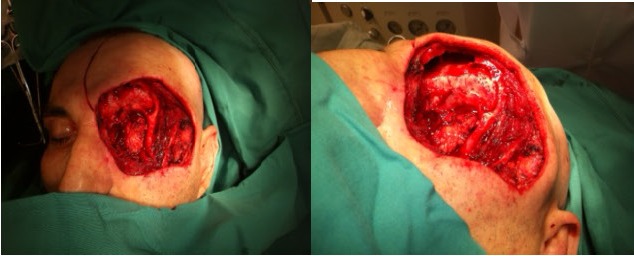
Post excisional defect

**Fig. 6:**
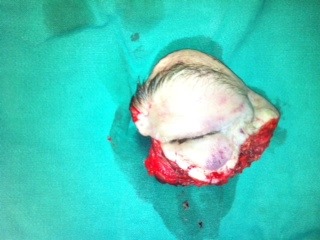
The excised piece

The surgical decision was orbital exenteration along with a radical excision of the frontal sinus (**Fig. 5**,**6**). The large post excisional cavity was covered with a temporal muscle flap. The skin defect was covered by rotation of a frontotemporal flap based on temporal superficial artery.

**Fig. 7,8:**
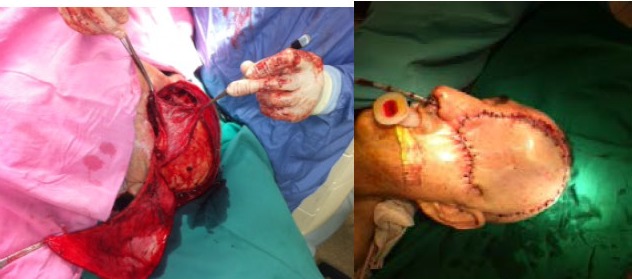
The cavity resulted after exenteration was covered by means of a temporal muscle flap; the skin defect was compensated by the rotation of a fronto-temporal flap

**Fig. 9:**
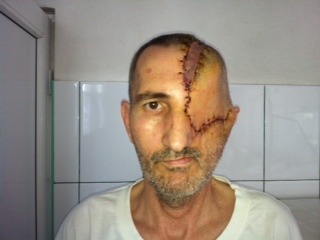
Result: five days after surgery

**Fig. 10:**
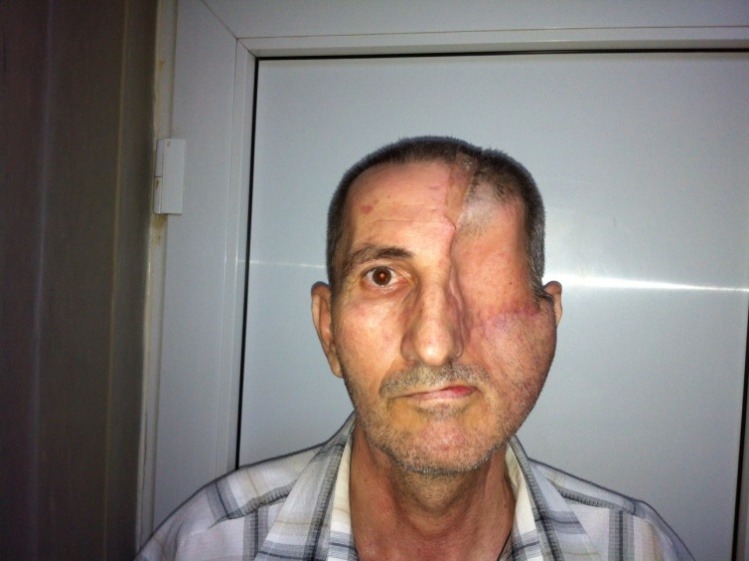
Five years result with no local recurrence and metastasis-free

## Discussion

BSCC is a rare and a high-grade histological variant of squamous cell carcinoma, which predominantly occurs in men in their 60 and 70s. There have been some reports of it being associated with tobacco and alcohol abuse [**2**,**10**,**20**]. It arises in a variety of anatomic sites, most frequently in the upper aero-digestive tract with strong predilection for the base of the tongue, supraglottic larynx and hypopharynx [**1**–**14**], but is also found in the anus, thymus and uterine cervix.

Since their report, the BSCCs of the other head and neck regions [**2**], such as the oral cavity, the palate, floor of the mouth, nasopharynx, and oropharynx, have been reported. Although this tumor type is most commonly found in the head and neck region, BSCC in the nasal cavity or in the paranasal sinuses is rare, with only 18 reported cases [**2**]. The case reported here is consistent with previous reports. Though the chief complaint in our case was cheek swelling, the most commonly reported clinical symptom of nasal or paranasal BSCC is unilateral nasal obstruction [**18**].

It has been reported that BSCC often shows an aggressive biologic behavior characterized by a high incidence of cervical lymph node metastasis and distant spread. In two reviews of the literature about BSCC in the head and neck, the incidences of neck node and distant metastasis are reported to be 64% and 44%, respectively, with 38% mortality at 17 months median survival [**2**,**12**]. Results of a case-control study by Soriano E et al [**21**] found a six times higher risk of distant metastasis compared to the usual type of SCC.

The midface defects, the ones concerning the orbital region, are the most challenging. They may be restricted to the orbital floor or be extended to the lateral and medial wall; sometimes, we must compensate a defect following the exenteration and excision of the paranasal sinuses. In the latter situation, we had to reconstruct the lost bony structures (with calvarial, rib, iliac free grafts) and the soft tissues thereafter or to perform a free transfer (a fibular or radial flap).

Sometimes, in oncological surgery, the tumoral aggressiveness associated with the patient’s poor general condition precludes the use of free transfer, which has proved to give the best aesthetic result and we only had the choice of loco-regional flaps.

The treatment of choice is a complete surgical excision supplemented by radiotherapy/ adjuvant chemotherapy. Although chemotherapy was suggested by some authors, because of the high incidence of distant metastasis and the relatively poor prognosis [**4**,**12**,**18**], a standard chemotherapy regimen for BSCC has not been established yet.

## Conclusions

We reported an additional case of BSCC of the maxillary sinus. Surgery with radiotherapy is currently the treatment of choice. Considering the high incidence of distant metastasis, further studies will be necessary to determine the effectiveness of chemotherapy.

The tumors in the orbital and periorbital area are a surgical challenge because of their aggressiveness; most of them are discovered in the advanced stages that make the surgical treatment more complicated.

In many cases, the defects after the surgical excision are large and the reconstructive plan must take into account the patient’s general status and the necessity of complementary oncologic treatments.
